# Improving Hydrophilicity and Inducing Bone-Like Apatite Formation on PPBES by Polydopamine Coating for Biomedical Application

**DOI:** 10.3390/molecules23071643

**Published:** 2018-07-05

**Authors:** Chengde Liu, Yizheng Li, Jinyan Wang, Cheng Liu, Wentao Liu, Xigao Jian

**Affiliations:** 1 State Key Laboratory of Fine Chemicals, Dalian University of Technology, Dalian 116024, China; liucd@dlut.edu.cn (C.L.); wangjinyan@dlut.edu.cn (J.W.); 2 Department of Polymer Science & Engineering, Dalian University of Technology, Dalian 116024, China; liyizheng@mail.dlut.edu.cn (Y.L.); lwt1122@mail.dlut.edu.cn (W.L.); 3 Liaoning Province Engineering Research Centre of High Performance Resins, Dalian 116024, China

**Keywords:** polydopamine, bone-like apatite, phthalazinone, PPBES, coating, cytocompatibility

## Abstract

Copoly(phthalazinone biphenyl ether sulfone) (PPBES) as a commercially available polyarylether is a promising orthopaedic implant material because its mechanical properties are similar to bone. However, the bioinert surface of polyarylether causes some clinical problems after implantation, which limits its application as an implant material. In this study, the surface of PPBES was modified by a biomineralization method of polydopamine-assisted hydroxyapatite formation (pHAF) to enhance its cytocompatibility. Polydopamine (PDA) coating, inspired by the adhesion mechanism of mussels, can readily endow PPBES with high hydrophilicity and the ability to integrate via the bone-like apatite coating. PPBES and PDA-coated PPBES were evaluated by scanning electronic microscopy (SEM), X-ray photoelectron spectroscopy (XPS), and contact angle measurement. The water contact angles were reduced significantly after coating with PDA. PDA was successfully synthesized on PPBES and more PDA was obtained by increasing the temperature. Bone-like apatite on PPBES (apatite-coated PPBES) was confirmed by SEM and transmission electron microscopy (TEM). The cytotoxicity of pristine PPBES and apatite-coated PPBES were characterized by culturing of NIH-3T3 cells. Bone-like apatite synthesized by pHAF could further enhance cytocompatibility in vitro. This study provides a promising alternative for biofunctionalized PPBES with improved cytocompatibility for bone implant application.

## 1. Introduction

Polyarylethers have been widely applied for biomedical application due to their chemical resistance, mechanical properties similar to bone, radiolucency, and their structures. Poly(aryl ether ether ketone) (PEEK), as a commercially-used biomaterial, has become a leading thermoplastic candidate for medical implant materials in orthopedics [1]. Bioactive materials as the surface coating or composite filler are used to improve its osteointegration [2,3,4,5,6]. Although composites can promote bioactivity, the drawbacks, including low implant strength and high roughness, retard its application as a biomaterial. 

The modification of polymer surfaces is another area of academic research to improve bone–implant interfaces. Ti and hydroxyapatite (HA) have been coated on PEEK to improve the bone–implant interface [7,8,9,10]. The authors reported that Ti-coated polyarylether implants improve osteogenesis compared to uncoated implants. HA coating on PEEK typically exhibits excellent biocompatibility and bioactivity in vitro and in vivo [11,12]. HA (chemical formula Ca_10_(PO_4_)_6_(OH)_2_) is most widely used to coat PEEK to enhance biocompatibility and osteogenesis because it is similar to bone mineral. HA coating can be fabricated on PEEK or PEEK composite using a thermal plasma spray coating [8], a cold spray technique [13], a spin coating technique [14], RF magnetron sputtering [15,16], aerosol deposition (AD) [17], and biomimetic mineralization [18,19]. Among the existing approaches, biomimetic mineralization is attractive due to its simplicity.

Biomimetic mineralization is to fabricate mimetic materials similar to nature’s sophisticated structures or their biologic functions [20]. A simple way to induce apatite coating called polydopamine-assisted hydroxyapatite formation (pHAF) has been found by a mussel-inspired strategy [21,22]. Dopamine polymerization is carried out in an aqueous solution to produce adhesive polydopamine (PDA) on various substrates. Catechol groups of PDA exhibit high calcium-ion coordination ability in simulated body fluid (SBF) and enhance hydroxyapatite formation on various substrates. In addition, the hydrophobic surface constrains the biomimetic mineralization. PDA, as a hydrophilic modifier, improves the wettability of the chitosan-based substrates to enhance its biomineralization capacity [23,24]. The effective and simple pHAF approach could be promising for the surface modification of PEEK.

**Figure 1:**
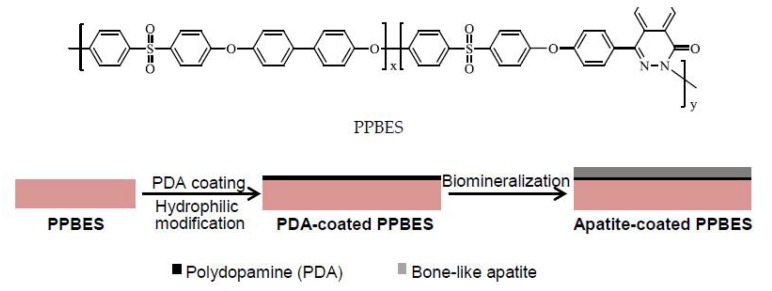
Chemical structure of Copoly(phthalazinone biphenyl ether sulfone) (PPBES) and schematic illustration of dopamine polymerisation and apatite biomineralisation on PPBES.

Copoly(phthalazinone biphenyl ether sulfone) (PPBES), a commercially-used polyarylether, has a structure similar to PEEK. Furthermore, the twisted and non-coplanar heterocyclic phthalazinone moiety is introduced into the main chain of PPBES (Figure 1), which results in PPBES with superior solubility in a variety of solvents (such as NMP, DMF, DMAc, and CHCl_3_) [25]. Moreover, the wholly aromatic and heterocyclic structure imparts PPBES with excellent mechanical properties. Moreover, it has better solubility, higher Tg and lower cost than PEEK [26]. The bioinert surface of PPBES also inhibits its biomedical application. 

In this work, the pHAF approach combining mussel-inspired polydopamine coating and biomimetic mineralization in SBF was used to improve the cytocompatibility of PPBES (Figure 1). The polydopamine layer was first employed to modify the surface of PPBES. Then, the apatite coating was created by the PDA layer using a biomineralization process. Finally, the apatite that formed on PPBES was examined and its cytocompatibility was then investigated in vitro. The results described herein demonstrate that PPBES with a bone-like apatite coating holds promising potential for biomedical applications. 

## 2. Results and Discussion

### 2.1. Mussel-Inspired Hydrophilization

Polydopamine, as a bio-inspired polymer, has similar properties to the proteins secreted from mussels. Polydopamine, with the ability to adhere to most surfaces, has been used to increase the hydrophilicity of a surface in membrane science and biomedical application [27,28,29]. However, the detailed mechanism of PDA formation is still elusive [30]. In the proposed mechanisms, 5,6-dihydroxindole of polydopamine can adsorb onto the surface of various materials by hydrogen bonding [31]. The hydrophilicity of PDA coating enhances the performance of functional materials [29,30]. The effects of deposition conditions, including time and dopamine concentration, on the hydrophilicity of substrates have been investigated systematically in this study. While dopamine concentration was increased from 2 to 8 mg/mL, the PDA coating became unstable (data not shown). After dopamine was dissolved in Tris-HCl buffer, the colorless solution gradually turned black. PDA formed particles and aggregates in the aqueous solution during the oxidative polymerization process and adsorbed on the substrate. PDA particles adsorbed after a thin PDA coating was formed [32]. 

**Figure 2:**
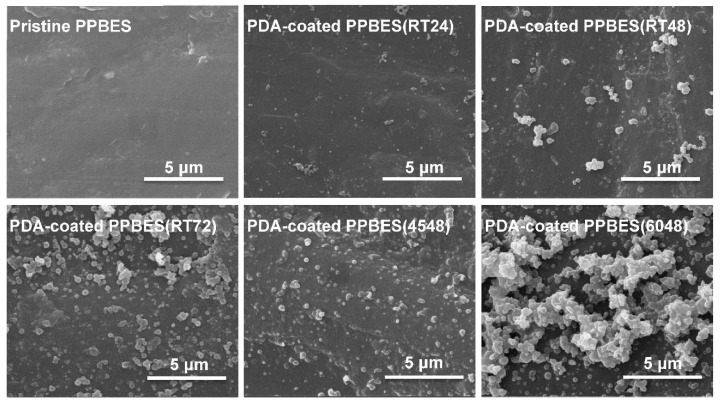
Surface morphology of the polydopamine (PDA)-coated PPBES.

PPBES plates were produced by hot pressing. The surface morphology changes were observed by SEM [33]. Pristine PPBES is smooth, while the roughness of PPBES was increased by the PDA nanoparticles after PDA coating (Figure 2). To obtain a stable and uniform PDA layer on PPBES, the dopamine solution was exchanged every 12 h. The growth of deposited PDA is influenced by reaction time and temperature [32]. PDA particles and aggregates on the PPBES plate were obtained after reacting for 72 h. It has been reported that the reaction rate for dopamine is highly elevated by reaction temperature. More PDA particles were observed on the PPBES plate when the reaction temperature was increased from room temperature to 60 °C. However, the coating became unstable and could be scratched easily.

**Figure 3:**
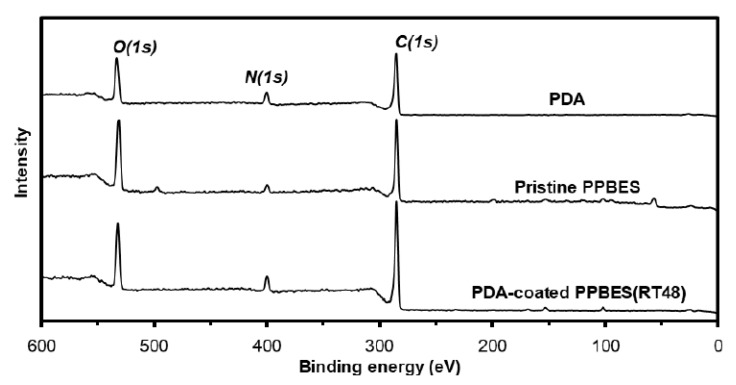
XPS survey spectra of PDA, PPBES, and PDA-coated PPBES.

XPS has been used to determine the elemental composition [34]. The XPS spectra acquired from PDA, pristine PPBES, and PDA-coated PPBES show the signals of O, N, and C, as shown in Figure 3. In detail, the nitrogen-to-carbon signal ratios (N/C) of PDA, pristine PPBES, and PDA-coated PPBES are 0.11, 0.06, and 0.08, respectively. The increase in the atomic composition of PDA-coated PPBES was observed compared to pristine PPBES, suggesting that PDA was coated on PPBES successfully. The difference of N/C between PDA and PDA-coated PPBES indicates that the XPS signals of PPBES was detected after PDA coating. 

**Figure 4:**
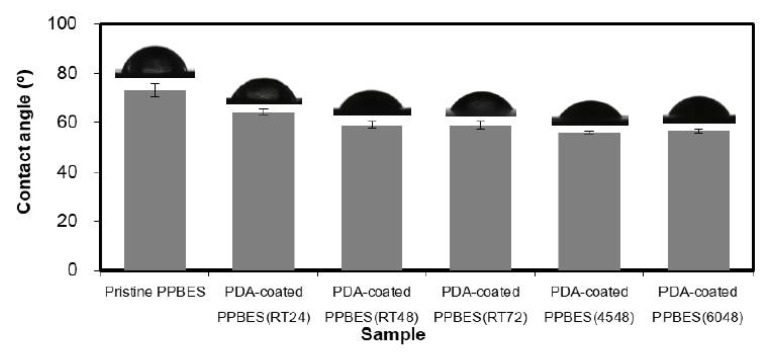
Water contact angles of pristine and PDA-coated PPBES prepared under different reaction conditions. PDA-coated PPBES(RT24) means that PDA coating was performed at room temperature for 24 h.

PDA coating is a simple and versatile method for surface modification towards functional materials. The hydrophilic-hydrophobic balance can be influenced by PDA deposition [35]. Contact angle measurement is a useful method of determining the surface hydrophilicity. The contact angles of these resultant samples are shown in Figure 4. After PPBES was modified by PDA coating, the static contact angles decreased obviously, which means improved hydrophilicity due to the formation of PDA layer on the PPBES plate. Moreover, the contact angle is also affected by the reaction time and temperature when preparing the PDA layer. As shown in Figure 4, PDA-coated PPBES(RT48) prepared at room temperature (RT) for 48 h demonstrated a smaller contact angle compared to PDA-coated PPBES(RT24) prepared at RT for 24 h. However, further extension of reaction time did not reduce the contact angle, as shown for PDA-coated PPBES(RT72). Another important factor of dopamine polymerization is reaction temperature. The contact angle decreased slightly as the reaction temperature was increased from RT to 45 °C. A little increase was observed from 45 to 60 °C, due to the rougher surface of the PDA layer that can increase the contact area with water. 

The surface energy (SE) data of PDA coating on PPBES versus reaction temperature and time are tabulated in Table 1. The SE value of the PPBES plate was apparently increased from 38.3 to 43.1 mN/m by the PDA coating, owing to the strong polar interactions in the PDA coating. The total SE increased from 43.1 to 43.9 mN/m by increased reaction time from 24 to 48 h and then slightly decreased with longer reaction time. Temperature influences the dopamine polymerization to a certain extent. The roughness of PDA-coated PPBES increased with elevating reaction temperature from 45 to 60 °C, which decreased the total SE slightly. Therefore, stable PDA coating was efficiently obtained at room temperature for 48 h and PDA-coated PPBES(RT48) was used for biomineralization. 

### 2.2. Characterization of Apatite-Coated PPBES

**Figure 5:**
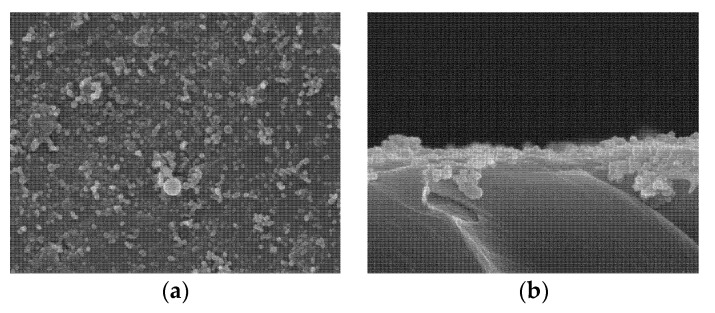
Apatite formation on PPBES via pHAF. (**a**) Top-down SEM image; (**b**). Cross-sectional SEM image.

Catecholamine moieties in the PDA coating can induce formation of hydroxyapatite crystals similar to natural hydroxyapatite in mineralized tissues on various materials [21]. Pristine PPBES did not facilitate apatite formation (data not shown). Quantities of small spherical particles were seen on PDA-coated PPBES after PDA coating (Figure 2). The hemispherical apatite particles adhered to the PDA coating and some clusters of apatite particles appeared on PDA-coated PPBES after biomimetic mineralization for 6 days (Figure 5). The apatite coating has a typical form for hydroxyapatite as reported by Ref. [36]. Moreover, the SEM images of the cross-sections of the PPBES plate modified with apatite coating are presented in Figure 5b. The thickness of the apatite coating is less than 1 µm.

**Figure 6:**
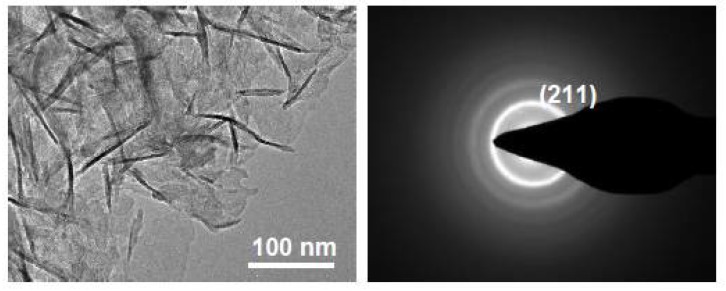
TEM and SEAD images of the bone-like apatite layer.

The morphology of the apatite layer was evaluated by TEM (Figure 6). Apatite coating on PPBES displayed characteristic particles similar to synthesized HA [37] and HA in bone tissue [38]. The bone-like apatite layer formed on PPBES was also confirmed by SEM data. Selected area electron diffraction (SAED) patterns were utilized to further verify the chemical composition of the apatite coating. The concentric ring patterns could be assigned to the (211) plane which corresponds to HA (Figure 6). 

### 2.3. Cell Viability and Adhesion

**Figure 7:**
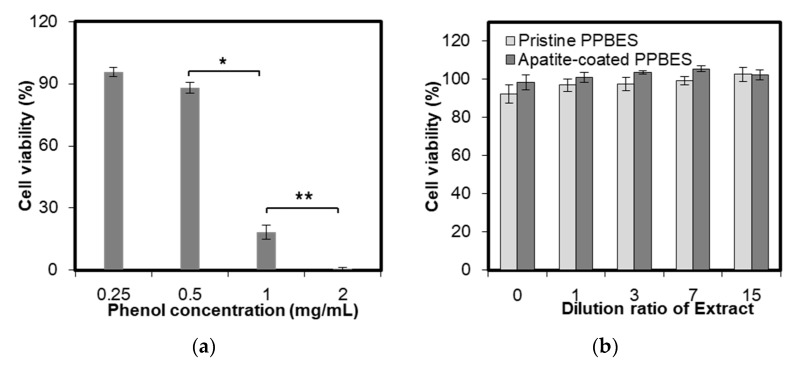
Cell viability incubated with phenol solution (**a**) and known concentrations of the aqueous-based extracts from pristine PPBES and apatite-coated PPBES (**b**). * and **: *p* < 0.05 relative to the phenol concentration 1 mg/mL.

The cytotoxicity of pristine PPBES and apatite-coated PPBES was characterized by culturing NIH-3T3 cells with the sample extracts. Phenol was used as positive control. It was observed that phenol elicited a cytotoxic response against NIH-3T3 cells when its concentration was increased to 1 mg/mL (Figure 7a). The aim of the extraction procedure based on the ISO 10993 protocol is to determine the cytotoxicity of the samples without affecting the mechanical properties of chemical compositions of the samples. MTT assay was used to quantify the toxicological hazard of the samples. Aqueous extracts of pristine PPBES and apatite-coated PPBES do not elicit a significant cytotoxic response against NIH-3T3 cells. The cell viability was increased slightly after the extracts were diluted with fresh cell culture medium. The apatite-coated PPBES is expected to be biosafe as an implant biomaterial.

**Figure 8:**
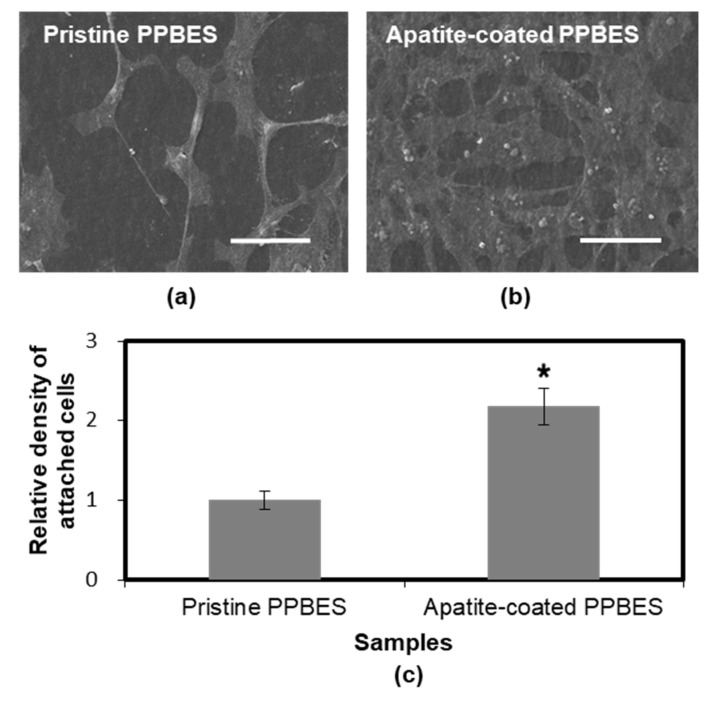
SEM images of NIH-3T3 cells cultured for 48 h on pristine PPBES (**a**) and apatite-coated PPBES (**b**) (scale bar = 50 μm). (**c**) Relative cell-adhesion density of NIH-3T3 cells cultured for 48 h on pristine PPBES and apatite-coated PPBES. * *p* < 0.05 relative to pristine PPBES.

Cell adhesion is responsible for cell functions and formation of new tissues [39]. Cell-adhesion properties were characterized by culturing NIH-3T3 cells with the two samples. The surface roughness and wettability of polymer substrates influence cell adhesion behavior significantly [40]. The water contact angle of apatite-coated PPBES (30.4 ± 2.9°) was decreased by the bone-like apatite coating compared to the pristine PPBES. The roughness was increased by the bone-like apatite coating (Figure 5). In addition, cell adherence to substrates can be facilitated by Ca^2+^ ions from apatite-coated substrates. After cell culture for 48 h, the samples with adhered cells were washed with PBS solution to remove the loosely attached cells. Figure 8 shows the representative SEM images of NIH-3T3 cells adhering to pristine PPBES and apatite-coated PPBES surfaces. The NIH-3T3 cells attached and proliferate to some extent on pristine PPBES surface. However, the cell-adhesion property of pristine PPBES is not good. Polymer substrates can be surface-modified to enhance cell-adhesion properties [41]. Cells on apatite-coated PPBES spread better than those on pristine PPBES and the cell area was high. Cells exhibited a more flattened morphology. Attached cells were increased significantly after pristine PPBES was coated with apatite. The relative cell-adhesion density on both of the two samples is shown in Figure 8. The density change is consistent with the result drawn from SEM images of attached cells. In conclusion, cell-adhesion property of PPBES is improved by apatite coating. 

## 3. Materials and Methods 

### 3.1. Materials

PPBES was supplied by Dalian Polymer New Material Co. Ltd. (Dalian, China). Dopamine hydrochloride and tris(hydroxymethyl)aminomethane (Tris) were purchased from Aladdin Corp. (Shanghai, China). MTT reagents were purchased from Sangon Biotech (Shanghai, China). 

### 3.2. Dopamine Polymerization and Biomineralization

PPBES plates were prepared by hot pressing and cleaned ultrasonically in acetone, ethanol, and deionized water successively. A series of PDA-coated PPBES samples were prepared by creating a polydopamine coating through dopamine polymerization on PPBES plates in 10 mM Tris buffer at pH 8.5. PDA-coated PPBES was extensively rinsed with deionized water and dried in a stream of N_2_ gas. PPBES coated by PDA at room temperature for 48 h (PDA-coated PPBES(RT48)) was chosen for biomineralization. It was transferred into 1.5× SBF and incubated at 37 °C. The composition of the 1.5× SBF was as follows (mM): Ca^2+^, 3.8; Na^+^, 213.0; K^+^, 7.5; Mg^2+^, 2.3; Cl^−^, 221.7; HCO^3−^, 6.3; HPO_4_^2−^, 1.5; SO_4_^2−^, 0.8. The 1.5× SBF was renewed every 24 h in order to preserve its ion concentration. After immersion for 6 days, apatite-coated PPBES was rinsed with deionized water and dried by N_2_ gas. 

### 3.3. Structural and Morphological Characterizations

The morphological changes after dopamine polymerization and biomineralization were investigated by scanning electron microscopy (SEM, QUANTA 450, FEI Company, Hillsborough, OR, USA) [42]. The samples were coated with platinum using a sputter coater (Quorum Technologies Ltd., East Sussex, UK).

Surface chemical compositions were analyzed using an X-ray photoelectron spectroscopy (XPS, ESCALABTM 250Xi, Thermo Fisher Scientific Inc., Waltham, MA, USA). AlKα X-ray was used as source. All binding energies were referenced to the C 1s component set to 285 eV.

The surface hydrophilicity and SE of the PPBESK layer and the coatings were characterized by a contact angle analyzer (JC2000D2W, Shanghai Zhongchen Digital Technic Apparatus Co. Ltd., Shanghai, China). Five measurements were performed for each sample at 25 °C and 55% relative humidity. The SE value was determined according to the Owens, Wendt, Rabel, and Kaelble (OWRK) method by using water and diiodomethane.

The apatite coating was confirmed by a Tecnai F30 transmission electron microscopy (TEM, FEI Company, Hillsborough, OR, USA) at an acceleration voltage of 300 kV. For TEM and selected-area electron diffraction (SAED) analyses, apatite coating was dispersed into ethanol by scraping and the suspension was dropped onto carbon-coated copper grids and dried by N_2_ gas before observation.

### 3.4. In Vitro Cell Culture

NIH-3T3 cells were seeded in Dulbecco’s modified eagle medium (DMEM) supplemented with 10% fetal bovine serum and penicillin/streptomycin at 37 °C under a humidified 5% CO_2_ atmosphere. The cell viability was determined using a 3-[4,5-dimethylthiazol-2-yl]-2,5-diphenyl tetrazolium bromide (MTT) assay [43,44]. Sterile pristine PPBES and apatite-coated PPBES were incubated with the complete culture medium in the ratio of 3 cm^2^ mL^−1^ between the surface area of the sample and the volume of cell culture medium according to the protocol ISO10993. The extract from the sample was aseptically diluted at volume ratios of 1:0, 1:1, 1:3, 1:7, and 1:15 using fresh culture medium. Cells were seeded at a density of 5 × 10^4^ cells mL^−1^ into wells in 96-well plates containing 100 μL of culture medium for 1 day. Then, the medium was exchanged with the respective extracts. Phenol solution and fresh culture medium were used as positive control and negative control, respectively. 

The cell-adhesion characteristics were assessed by cell culture on pristine and apatite-coated PPBES. The surfaces after incubation were washed with PBS solution to remove the loosely attached cells. Fixation with 4% glutaraldehyde for 2 h and dehydration in a series of ethanol solutions (50–100%) were carried out for cell imaging. The cell immobilized PPBES and HA-coated PPBES were imaged using a scanning electron microscope. Prior to the SEM measurements, specimens were fixed on the holders and sputtered with a thin Pt layer. For cell number determination, the surfaces were incubated with 0.25% trypsin solution for 5 min at 37 °C to detach the cells. The detached cells were collected and counted using a hemocytometer.

### 3.5. Statistical Analysis 

The data were shown as the means ± standard deviation for each group. A one-way analysis of variance (ANOVA) was utilized to perform the statistical analysis among the groups. *p*-value < 0.05 was regarded as statistically significant. 

## 4. Conclusions

The low bioactivity of current synthetic polyarylethers, such as PEEK, has limited the choice of materials, slowing down the development of bone implant materials. PPBES is a kind of commercial polyarylether which has the potential to replace PEEK as an implant material. In this study, it was demonstrated that the pHAF method was a feasible method to produce a novel apatite coating on synthetic PPBES. The hydrophilic modification and catecholamine moieties are beneficial for biomineralization. In vitro studies, including MTT assay and cell adhesion tests, indicated that the apatite coating on PPBES did not elicit cytotoxicity against NIH-3T3 cells and promoted cell adhesion significantly. As the pHAF approach can be used to integrate hydroxyapatites onto virtually any material morphology, it is an attractive approach to prepare functional coatings on polyarylether medical implants.

## Figures and Tables

**Table 1:** Effect of Reaction Time and Temperature on the SE of PDA-coated PPBES.

Sample ^a^	Contact Angle (deg)		Surface-Energy Components (mN/m)		
	Water	Diiodomethane	σ_s_^a^	σ_s_^b^	σ_s_^c^
Pristine PPBES	73.2 ± 2.7	48 ± 1.2	38.3	28.4	9.9
PDA-coated PPBES(RT24)	64.3 ± 1.0	44.7 ± 0.8	43.1	28.1	15.0
PDA-coated PPBES(RT48)	59.0 ± 1.3	52.9 ± 1.4	43.9	22.4	21.5
PDA-coated PPBES(RT72)	58.9 ± 1.5	53.9 ± 1.8	43.8	22.9	17.8
PDA-coated PPBES(4548)	56.0 ± 0.5	52.7 ± 1.6	45.9	22.0	23.9
PDA-coated PPBES(6048)	56.7 ± 0.7	60.0 ± 0.6	44.2	18.1	26.1

σ_s_^a^: total SE; σ_s_^b^: disperse part of SE; σ_s_^c^: polar part of SE.
